# Alginate-Chitosan Coated Nanoliposomes as Effective Delivery Systems for Bamboo Leaf Flavonoids: Characterization, In Vitro Release, Skin Permeation and Anti-Senescence Activity

**DOI:** 10.3390/antiox11051024

**Published:** 2022-05-23

**Authors:** Yanpei Gu, Zhenlei Zhao, Fan Xue, Ying Zhang

**Affiliations:** Zhejiang Key Laboratory for Agro-Food Processing, Zhejiang Engineering Center for Food Technology and Equipment, College of Biosystems Engineering and Food Science, Zhejiang University, Hangzhou 310058, China; yanpeigu@zju.edu.cn (Y.G.); zhenlei-zhao@foxmail.com (Z.Z.); xf21713038@zju.edu.cn (F.X.)

**Keywords:** bamboo leaf flavonoids, nanoliposome, biopolymer conjugation, in vitro release, skin permeability, anti-senescence activity

## Abstract

The use of bamboo leaf flavonoids (BLF) as functional food and cosmetic ingredients is limited by low bioavailability and difficulty in being absorbed by the intestine or skin. The aim of this study was to prepare BLF-loaded alginate-chitosan coated nanoliposomes (AL-CH-BLF-Lip) to overcome these challenges. The nanocarriers were characterized by dynamic light scattering, high performance liquid chromatography, Fourier transform infrared spectroscopy and differential scanning calorimetry. The biological activity was analyzed by in vitro antioxidant activity, transdermal absorption, cytotoxicity and AAPH induced HaCaT cell senescence model. The results showed that the size of nanocarriers ranged from 152.13 to 228.90 nm and had a low polydispersity index (0.25–0.36). Chitosan (CH) and alginate (AL) were successfully coated on BLF-loaded nanoliposomes (BLF-Lip), the encapsulation efficiency of BLF-Lip, BLF-loaded chitosan coated nanoliposomes (CH-BLF-Lip) and AL-CH-BLF-Lip were 71.31%, 78.77% and 82.74%, respectively. In addition, BLF-Lip, CH-BLF-Lip and AL-CH-BLF-Lip showed better in vitro release and free radical scavenging ability compared with naked BLF. In particular, the skin permeability of BLF-Lip, CH-BLF-Lip, and AL-CH-BLF-Lip increased 2.1, 2.4 and 2.9 times after 24 h, respectively. Furthermore, the use of nanoliposomes could significantly improve the anti-senescence activity of BLF (*p* < 0.01). Conclusively, alginate-chitosan coated nanoliposomes are promising delivery systems for BLF that can be used in functional foods and cosmetics.

## 1. Introduction

Flavonoids are a class of secondary metabolites widely existing in nature and that have a variety health and medicinal values. The main active components of bamboo leaf flavonoids (BLF) isolated from bamboo leaves are four carbon glycoside flavonoids, namely orientin, isoorientin, vitexin and isovitexin (Supplementary Figure S1) [1]. Studies have shown that BLF have a variety of biological effects such as antioxidant, lipid-lowering, anti-radiation, anti-bacterial, anti-inflammatory and anti-aging, and have a broad application prospect in functional foods and cosmetics [2,3,4,5,6,7]. However, similar to other flavonoids, BLF have a darker color, are difficult to be absorbed, and are sensitive to factors such as light, oxygen and high temperature, which limit their applications. The use of nano carriers can effectively improve the stability and bioavailability of the embedded compounds [8,9]. Among them, nanoliposomes have been widely used due to the advantages of sustained release, cell affinity, histocompatibility, low toxicity and targeting. Usually composed of natural phospholipids and cholesterol, nanoliposomes have one or more closed vesicles with lipid bilayer structure that can encapsulate lipophilic and hydrophilic compounds, in which hydrophobic compounds are inserted into lipid bilayer membranes and hydrophilic compounds are encapsulated in the internal aqueous phase [10,11]. However, nanoliposomes have low physical stability, and are easily affected by light, acid, alkali and other factors during long-term storage, resulting in aggregation and fusion of vesicles, and ultimately lead to the leakage of embedded compounds. The oxidation and hydrolysis of phospholipids is one of the main reasons for the chemical instability of nanoliposomes [12,13].

Layer by layer self-assembly refers to the formation of self-assembled multilayers by alternating deposition in polyelectrolyte solutions with opposite charges [14,15]. Studies have shown that the biopolymer coated nanoliposomes prepared by layer-by-layer self-assembly can reduce oxidative damage and hydrolysis reaction. In addition, an electrostatic bridge can be formed between phospholipid and biopolymer to minimize the permeability of the phospholipid bilayer, thereby improving the stability of nanoliposomes [10,16]. At present, a large number of studies on biopolymer coated nanoliposomes have been reported.

Chitosan (CH) is a cationic polysaccharide obtained by deacetylation of chitin. Under acidic condition, positively charged CH binds to negatively charged liposomes through electrostatic interaction. At the same time, the residual acyl groups of CH can be inserted into the phospholipid membrane bilayer of liposomes [17,18]. Shishir et al. [18] found that CH coated nanoliposomes had higher stability, better sustained-release and improved cellular uptake. Alginate (AL) is a non-toxic inert polyanionic polysaccharide that is widely used as dietary fiber, thickener, emulsifier and stabilizer in food industry. Liu et al. [16,19] found that AL-CH-coated nanoliposomes had higher physical and digestive stability compared with uncoated nanoliposomes. Cui et al. [20] proved that AL and CH could improve the stability and transmembrane transport efficiency of chito-oligosaccharides. In addition, CH-AL nanogel carriers were able to increase the skin penetration and sustained release of pirfenidone [21].

In this study, we used the thin layer dispersion method combined with sonication to prepare BLF-loaded nanoliposomes (BLF-Lip), and further prepared biopolymer coated BLF-Lip by electrostatic layer by layer self-assembly. The carrier systems were characterized by dynamic light scattering (DLS), high performance liquid chromatography (HPLC), Fourier transform infrared (FTIR) spectroscopy and differential scanning calorimetry (DSC). We studied the effects of encapsulation of BLF on skin permeability and in vitro release. In addition, changes in antioxidant activity and anti-senescence activity were also evaluated when encapsulating BLF. The above research aimed to improve the bioavailability, transdermal absorption and anti-senescence activity of BLF, and expand their application value in functional foods and cosmetics.

## 2. Materials and Methods

### 2.1. Materials

Bamboo-leaf flavonoids (the mass fraction of four carbon glycoside flavonoids was 80.66%, Supplementary Figure S2) were prepared in laboratory. L-α-Phosphatidylcholine, cholesterin, CH, AL, 2–2′azino-bis-(3-ethylbenzothiazoline-6-sulfonic acid) diammonium salt (ABTS), 2,2-Diphenyl-1-picrylhydrazyl (DPPH), 2,2′-Azobis (2-methylpropionamidine) dihydrochloride (AAPH), 3-(4,5-dimthyl-2-thiazolyl)-2,5-diphenyl-2-H-tetrazolium bromide (MTT), 4′,6-diamidino-2-phenylindole (DAPI) were obtained from Sigma Aldrich (St. Louis, MO, USA). Reagents required for cell culture were purchased from HyClone (Logan, UT, USA) and Gibco (Grand Island, NY, USA), respectively. K9M-H3 and Alexa Fluor 488-conjugated Goat Anti-Rabbit IgG (H + L) antibodies were purchased from ABclonal Technology (Wuhan, Hubei, China); p21, p16 and Goat Anti-Rabbit secondary antibodies were obtained from Cell Signaling Technology (Beverly, MA, USA). HPLC-grade methanol was obtained from TEDIA (Cincinnati, OH, USA). The 10 kDa dialysis bag was provided by Spectrum (San Jose, CA, USA). Other chemicals and reagents were purchased from Sinopharm (Shanghai, China).

### 2.2. Preparation of BLF-Lip

The thin layer dispersion method combined with sonication was used to prepare BLF-Lip [18,19]. First, phosphatidylcholine, cholesterol, Tween-80 and vitamin E were dissolved in absolute ethanol at a mass ratio of 6:1:1:8:0.12 and stirred evenly with a magnetic stirrer. Then, the ethanol was removed under vacuum at 55 °C by a rotary evaporator. After the dried lipid film was formed, we slowly added phosphate buffer solution (PBS; pH 7.4, 0.05 M) dissolved with 1.5 mg/mL BLF. Then after being fully washed for 1 h under low-speed rotation condition, the primary BLF loaded liposomes with lipid concentration of 8 mg/mL were obtained. The primary BLF loaded liposomes were sonicated using SK8210HP sonicator (Kudo, Shanghai, China) for 8 min (75% of ultrasonic time set) at a power of 120 W to obtain BLF-Lip. The whole preparation process was protected from light, and the obtained BLF-Lip were stored at 4 °C under dark condition.

### 2.3. Preparation of Biopolymer Coated Nanoliposomes

A total of 0.1 g CH and AL were dissolved in 100 mL of 1% glacial acetic acid solution and deionized water, respectively, stirred overnight and then had the pH adjusted to 5.5. After centrifugation and filtration, 0.1% CH and AL solution were obtained. CH and AL were conjugated to the surface of BLF-Lip in two steps according to the layer by layer self-assembly approach [16]. In short, the above BLF-Lip were placed in a burette and added dropwise to 0.1% CH solution (1:1, *v*/*v*), incubated for 1 h under gentle stirring to fully deposit CH on the surface of BLF-Lip, then adjusted pH to 5.5. The same procedure was used to add the positively charged BLF-loaded chitosan coated nanoliposomes (CH-BLF-Lip) to the negatively charged AL solution, then adjusted pH to 5.5 to obtain BLF-loaded alginate-chitosan coated nanoliposomes (AL-CH-BLF-Lip). The solution was stored at 4 °C for further analysis.

### 2.4. Particle Size, Polydispersity Index (PDI) and Zeta Potential Measurement

AL-CH-BLF-Lip, CH-BLF-Lip and BLF-Lip were diluted 2.5, 5 and 10 times with ultrapure water, respectively. A Nano-ZS90 dynamic light scattering instrument (Malvern, United Kingdom) was used to measure the particle size, PDI and zeta potential at 25 °C, the scattering angle was 90°, the refractive index was 1.330, and the viscosity was 0.933. Each sample was determined at least three times in parallel.

### 2.5. Standard Curve and Encapsulation Efficiency (EE) Determination

The BLF were accurately weighed in a 10 mL flask, dissolved in methanol and prepared solutions of 400, 200, 100, 50, 25, 10, 5 μg/mL, respectively. HPLC with diode array detector (Dionex ultimate 3000, ThermoFisher Scientific, Waltham, MA, USA) was used to analyze the amount of BLF. Samples were injected into Luna C_18_ column (250 mm × 4.60 mm × 5 μm) after filtration with 0.22 μm membrane for analysis according to the following chromatographic conditions: scanning wavelength range of 200–400 nm, quantitative wavelength of 330 nm, column temperature of 40 °C, flow rate of 0.5 mL/min, injection volume of 10 μL, and mobile phase consisted of acetonitrile −0.1% trifluoroacetic acid (20:80, *v*/*v*). The standard curve was drawn by peak area normalization method.

The EE was determined by ultrafiltration centrifugation [22]. A 50% methanol aqueous solution containing 1% acetic acid was added to the nanoliposomes (10:1, *v*/*v*), sonicated for 10 min, and centrifuged at 10,000 rpm for 10 min to obtain total BLF. An equal amount of nanoliposomes were added to the inner tube of ultrafiltration tubes (MWCO 10 kDa, Millipore, Billerica, MA, USA), centrifuged at 4000 rpm for 40 min, and 1 mL PBS was added to repeat the above operation once. The free BLF was obtained in the outer tube of the ultrafiltration tube.

EE (%) = (Total amount of BLF − free amount of BLF)/Total amount of BLF × 100(1)

### 2.6. FTIR

In order to confirm that the biopolymers were coated on the surface of BLF-Lip, FTIR analysis was performed using an FTIR Avatar 370 spectroscopy (Nicolet, Madison, WI, USA). BLF, CH, AL and lyophilized BLF-loaded nanoliposomes were scanned at 4000 to 400 cm^−1^ with a resolution of 4 cm^−1^ using the KBr tablet method. OMNIC software version 8 was used to analyze data.

### 2.7. DSC

A total of 2–5 mg lyophilized BLF-loaded nanoliposomes were placed in an alumina crucible and put in the sample chamber of DSC (Mettler Toledo, Zurich, Switzerland). The blank alumina crucible was used as a reference, the heating rate was 10 °C/min, the nitrogen flow rate was 30 mL/min, the scanning temperature range was 20–250 °C, and the thermodynamic curve was recorded.

### 2.8. In Vitro Release

The dialysis method was used to measure the in vitro release of BLF and BLF-loaded nanoliposomes, mainly referring to the method of Lopes et al., with some modifications [23]. One mL of naked BLF, BLF-Lip, CH-BLF-Lip and AL-CH-BLF-Lip were placed in a 10 kDa dialysis bag, then suspended in 50 mL PBS (pH6.0), and stirred in a magnetic stirring apparatus at a speed of 100 rpm under 37 °C. One mL of the medium outside dialysis bag was sampled after 0.5, 1, 2, 4, 6, 8, 12 and 24 h, and replaced with the same volume of PBS at the same time. The cumulative release (CR) was calculated by the following formula.

(2) $$CR\left(\%\right)=\frac{C_{n}\times V+\sum_{i=1}^{n-1}C_{i}\times V_{i}}{C_{0}}\times 100.$$

$C_{0}$, $C_{i}$ and $C_{n}$ were the concentrations of BLF (μg/mL) measured at the 0, i and nth sampling, respectively. V and $V_{i}$ were the receiving liquid volume and sampling volume, respectively.

### 2.9. In Vitro Skin Permeation Study

The Franz diffusion cell method was used to determine the in vitro skin permeation [24]. C57BL/6J mice were anesthetized and sacrificed by neck amputation. The hair on the back skin of mice was removed with depilatory cream, and the connective tissue and subcutaneous fat were peeled off. The skin tissue was fixed on the diffusion cell, and 2 mL naked BLF and BLF-loaded nanoliposomes were added to the supply chamber. The effective diffusion area was 4.9 cm^2^, the volume of the receiving chamber was 7 mL, and the receiving solution was PBS (pH 7.4). During the whole experiment, the diffusion cell was stirred in a magnetic stirring apparatus at a speed of 100 rpm under 37 °C. A total of 0.4 mL receiving solution was sampled after 0.5, 1, 2, 4, 6, 8, 12 and 24 h, and the same volume of the receiving solution was supplemented at the same time. The cumulative permeation (CP) was calculated by the following formula.

(3) $$CP=\frac{C_{n}\times V+\sum_{i=1}^{n-1}C_{i}\times V_{i}}{A}.$$

$C_{i}$ and $C_{n}$ were the concentrations of BLF (μg/mL) measured at the i and nth sampling, respectively. A was the effective diffusion area. V and $V_{i}$ were the receiving liquid volume and sampling volume, respectively.

### 2.10. ABTS Radical Cation (ABTS^+^) Assay

The free radical scavenging activity of naked BLF and BLF-loaded nanoliposomes was studied by ABTS^+^ assay [18]. A total of 7.4 mM ABTS and 2.6 mM potassium persulfate solution were mixed in a volume ratio of 1:1 and incubated at room temperature in dark for 12 h to obtain ABTS^+^ stock solution, diluted with absolute ethanol to an absorbance at 734 nm of 0.70 ± 0.02 to obtain ABTS^+^ working solution. Then, 200 µL naked BLF and BLF-loaded nanoliposomes were mixed with 0.8 mL ABTS^+^ working solution, and incubated for 6 min at room temperature, the control group was replaced with the same volume of ethanol. The absorbance was measured at 734 nm using a bio-Tek microplate reader (Winooski, VT, USA).

ABTS scavenging capacity (%) = (A_control_ − A_sample_)/A_control_ × 100. (4)

### 2.11. DPPH Assay

DPPH assay followed the previously described procedure with some modifications [25]. One mL of 0.2 mmol/L DPPH prepared with anhydrous ethanol was shaken with 0.2 mL naked BLF and BLF-loaded nanoliposomes, and then incubated for 30 min at room temperature in dark. The absorbance was measured at 517 nm using a bio-Tek microplate reader (Winooski, VT, USA). The control group was replaced with the same volume of anhydrous ethanol, and the positive control was Trolox.

DPPH scavenging capacity (%) = (A_control_ − A_sample_)/A_control_ × 100. (5)

### 2.12. Cell Culture

HaCaT cells were obtained from the Cell Bank of the Chinese Academy of Sciences. The resuscitated HaCaT cells were cultured in DMEM high glucose medium containing 15% fetal bovine serum and 1% antibiotics (penicillin, streptomycin) and placed in a humidified incubator at 37 °C and 5% CO_2_. Approximately, 80% confluent HaCaT cells were digested with a 0.25% Trypsin/0.02% EDTA solution and passaged, and the logarithmic growth phase cells were taken for experiments.

### 2.13. Cytotoxicity Study

HaCaT cells were seeded into the 96-well cell culture plates at a density of 5 × 10^3^ cells/well. Naked BLF and BLF-loaded nanoliposomes of different doses were added after the cells were adherent overnight. At the same time, a blank group containing only the medium and a negative control group containing only cells were set. After being incubated for 24 h, we absorbed the supernatant and added the fresh medium containing 10 μL MTT solution to each well. After incubation in the incubator for 4 h, the culture medium was absorbed and shaken with DMSO solution for 10 min. The absorbance of each well was measured at a wavelength of 490 nm using a bio-Tek microplate reader (Winooski, VT, USA).

### 2.14. Anti-Senescence Activity

#### 2.14.1. Assessment of Cell Proliferation

In order to find the protective effect of naked BLF and BLF-loaded nanoliposomes on AAPH-induced senescence, HaCaT cells were cultured overnight and pretreated with samples for 4 h, then co-incubated with AAPH for 48 h. At the same time, a blank group containing only the medium and a negative control group containing only cells without AAPH and samples were set. The absorbance of each well was measured at a wavelength of 490 nm using a bio-Tek microplate reader (Winooski, VT, USA).

#### 2.14.2. Confocal Fluorescence Microscopy

To detect senescence-associated heterochromatin foci (SAHF) formation in cultured cells by immunofluorescence analysis, HaCaT cells were fixed and permeabilized at room temperature with 4% paraformaldehyde and 0.5% Triton X-100 solution, respectively. After washing with PBS for three times, cells were blocked by PBS containing 3% goat serum and 1% bovine serum albumin for 1 h, then followed by adding the prepared primary antibody specific for K9M-H3 and incubating at 4 °C overnight. Cells were then incubated with a secondary antibody specific for Alexa Fluor 488-conjugated Goat Anti-Rabbit IgG (H + L), and nuclei were marked by 1 µg/mL DAPI solution at the same time. Cells with condensed K9M-H3 that co-localized with DAPI in the nuclei were considered as SAHF positive, and the percentage of SAHF positive cells relative to the total number of cells was calculated.

#### 2.14.3. Western Blotting

RIPA Lysis buffer supplemented with protease inhibitors (Beyotime, Shanghai, China) was used to extract the total protein of each group of HaCaT cells. The protein concentration was quantified using the BCA protein assay kit (Beyotime, Shanghai, China). After separation by polyacrylamide gel electrophoresis, the protein was transferred to PVDF membrane. Blots were incubated with K9M-H3, p21 and p16 antibodies (1:1000), washed, and incubated with a horseradish peroxidase (HRP)-labeled secondary antibody. Visualizing the bands by using ECL commercial kit (Beyotime, Shanghai, China) for chemiluminescence. Taking GAPDH as the reference protein, Image J software was used to analyze the gray value to quantify the protein expression.

### 2.15. Statistical Analysis

All experiments were repeated at least three times, and the data were expressed as mean ± standard deviation (SD). Statistical analyses were performed by SPSS 19.0 (IBM, Chicago, USA), significant differences between the groups were determined by using a one-way analysis of variance (ANOVA), taking *p* < 0.05 as significant difference, and *p* < 0.01 as extremely significant difference.

## 3. Results and Discussion

### 3.1. Particle Size, PDI, Zeta Potential and EE

The characteristics of BLF-Lip, CH-BLF-Lip and AL-CH-BLF-Lip were summarized in Table 1. The particle size, zeta potential and PDI of BLF-Lip were 152.13 ± 5.20 nm, −3.81 ± 0.79 mV and 0.25 ± 0.06, respectively. The particle size of CH-BLF-Lip and AL-CH-BLF-Lip was increased to 194.63 ± 4.25 nm and 228.90 ± 4.89 nm, which preliminarily proved that biopolymers were coated on BLF-Lip. This phenomenon could be explained by the electrostatic interaction between the phospholipid head group PO_4_^−3^ and the specific functional groups NH_3_^+^ and COO^−^ of CH and AL. The charged nanoliposomes can reduce aggregation and fusion to improve stability. Zeta potential is an important indicator to measure the amount of charge [26]. The zeta potential of CH-BLF-Lip was 8.43 ± 1.56 mV, which changed to −27.77 ± 0.45 mV after coating AL. Alternating positive and negative charges further confirmed that CH and AL were successfully coated on the surface of BLF-Lip [27,28]. PDI defines the homogeneity of size dispersion in nanoparticles. Compared with BLF-Lip, the PDI value of CH-BLF-Lip and AL-CH-BLF-Lip had been increased to a certain extent, but was still less than 0.36, which could be considered as moderately homogeneous dispersion [28]. The EE detected by HPLC also confirmed that BLF was encapsulated in nanoliposomes. The EE of BLF-Lip was determined to be 71.31 ± 1.67%, increased to 78.77 ± 1.59% and 82.74 ± 0.75% after coating CH and AL, respectively. The possible reason for the increase of EE is that the free BLF are also encapsulated on the surface of nanoliposomes during the modification of polysaccharides. In addition, the dense bilayer formed by CH and AL on the surface of Lip prevents the fast release of BLF.

### 3.2. FTIR Analysis

**Figure 1:**
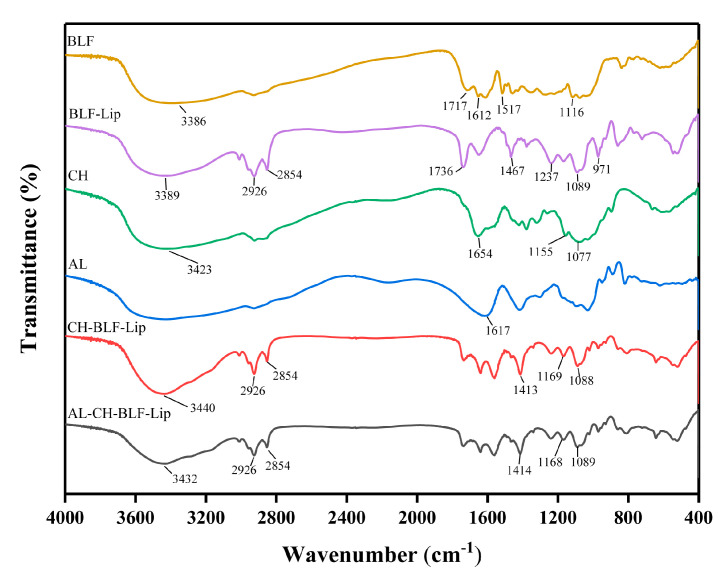
Fourier transform infrared (FTIR) spectra of bamboo leaf flavonoids (BLF), BLF−loaded nanoliposomes (BLF−Lip), chitosan (CH), alginate (AL) and BLF−loaded chitosan coated nanoliposomes (CH−BLF−Lip) and BLF−loaded alginate-chitosan coated nanoliposomes (AL−CH−BLF−Lip).

The FTIR spectra of BLF, BLF-Lip, CH, AL, CH-BLF-Lip and AL-CH-BLF-Lip were shown in Figure 1. The characteristic peaks of BLF were found at the wavenumbers of 3386 cm^−1^ (O-H stretching vibration), 1717 cm^−1^ (C=O stretching vibration), 1612 and 1517 cm^−1^ (the aromatic ring C=C skeleton stretching vibration) and 1116 cm^−1^ (C-O stretching vibration). The characteristic peaks of nanoliposomes mainly including 3389 cm^−1^ (O-H stretching of hydroxy bond of cholesterol in liposome and water vapor association during pressing), 2926 cm^−1^ and 2854 cm^−1^ (asymmetric and symmetric stretching vibration of C-H bonds in CH_2_), 1736 cm^−1^ (C=O stretching vibration in phospholipid), 1467 cm^−1^ (asymmetric stretching vibration of CH_3_), 1237 cm^−1^ and 1089 cm^−1^ (PO_2_^−^ symmetric and asymmetric stretching vibration) and 971 cm^−1^ (asymmetric stretching vibration of C-C-N^+^). After BLF were encapsulated in nanoliposomes, the FTIR spectrum of BLF-Lip was similar to nanoliposomes and showed almost no characteristic peaks of BLF. The result indicated that BLF might be well encapsulated in nanoliposomes.

The wavenumber at 3423 cm^−1^ (O-H stretching vibration), 1654 cm^−1^ (C=O stretching vibration), 1155 and 1077 cm^−1^ (C-O stretching vibration) were the mainly characteristic peaks of CH. The peaks of 1736 cm^−1^, 1467 cm^−1^ and 1237 cm^−1^ belonging to liposomes were shifted in CH-BLF-Lip and AL-CH-BLF-Lip, indicating the formation of new hydrogen bonds or the strengthening of hydrogen bonds. In addition, the 1654 cm^−1^ of CH and the 1617 cm^−1^ (carboxyl asymmetric stretching vibration) of AL disappeared. In summary, the alteration of characteristic peaks confirmed the existence of strong electrostatic interaction between AL, CH and BLF-Lip, thus forming AL-CH-BLF-Lip.

### 3.3. DSC Analysis

**Figure 2:**
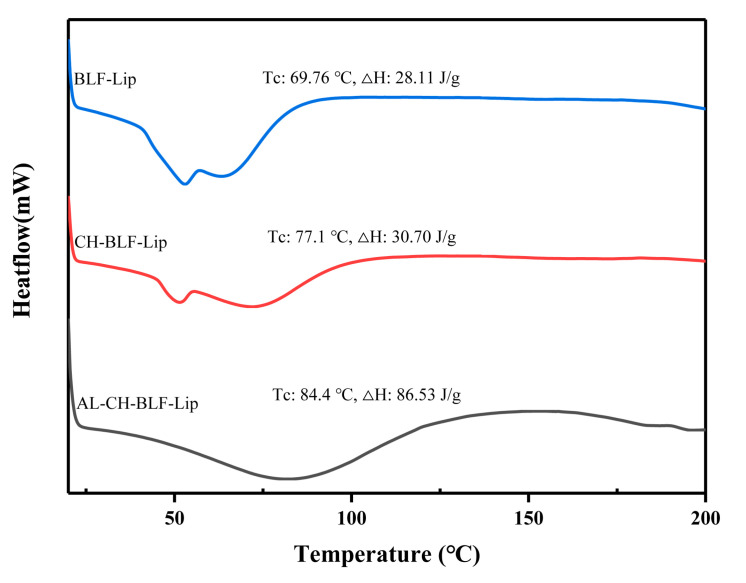
DSC analysis. BLF-Lip, Tc, Phase transition temperature; ΔH, Enthalpy variation. BLF-Lip, BLF-loaded nanoliposomes; CH-BLF-Lip, BLF-loaded chitosan coated nanoliposomes; AL-CH-BLF-Lip, BLF-loaded alginate-chitosan coated nanoliposomes.

**Figure 3:**
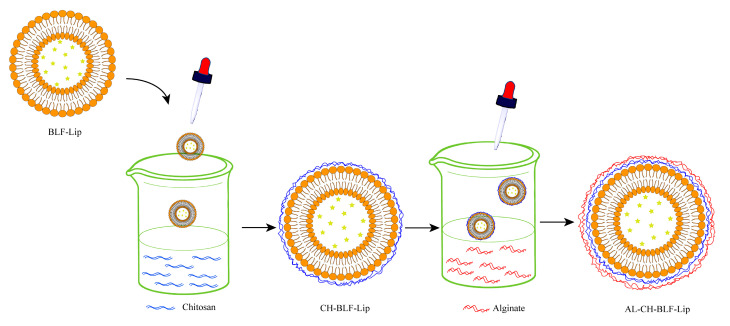
Illustration of the electrostatic deposition of chitosan and alginate layer onto the surface of nanoliposomes. BLF-Lip, BLF-loaded nanoliposomes; CH-BLF-Lip, BLF-loaded chitosan coated nanoliposomes; AL-CH-BLF-Lip, BLF-loaded alginate-chitosan coated nanoliposomes.

DSC was used to understand the effects of polysaccharides on the thermal behavior of BLF-Lip. Enthalpy energy (ΔH) and phase transition temperature (T_c_) were measured (Figure 2). BLF-Lip, CH-BLF-Lip, AL-CH-BLF-Lip showed fully wide endothermic peaks (69.76, 77.1 and 84.4 °C, respectively) during phase transition, indicating poor crystallinity of all samples. Compared with BLF-Lip, the T_c_ and ΔH of CH-BLF-Lip and AL-CH-BLF-Lip increased, which suggested that the layer-by-layer modification of polysaccharides improved the thermal stability of BLF-Lip. The reasonable explanation is that CH and AL affect the membrane fluidity of nanoliposomes, which is consistent with the results of other flavonoids [29]. Finally, based on the above results, a schematic illustration for the layer-by-layer deposition of CH and AL onto the surface of BLF-NL is shown in Figure 3.

### 3.4. Antioxidant Activity

**Figure 4:**
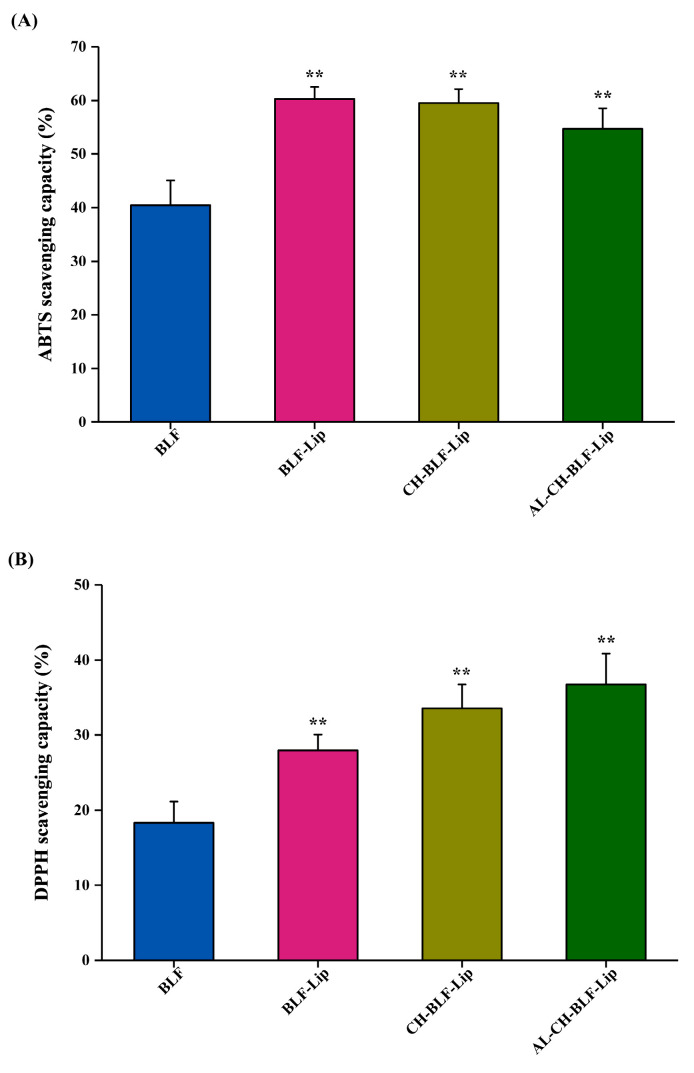
(**A**) ABTS radical scavenging capacity. (**B**) DPPH radical scavenging capacity. Data are presented as mean ± SD of three independent replications, ** *p* < 0.01 vs. BLF group. BLF, bamboo leaf flavonoids; BLF-Lip, BLF-loaded nanoliposomes; CH-BLF-Lip, BLF-loaded chitosan coated nanoliposomes; AL-CH-BLF-Lip, BLF-loaded alginate-chitosan coated nanoliposomes.

BLF are recognized as dietary antioxidants with a variety of biological activities. In this study, the antioxidant activity of naked BLF and BLF-loaded nanoliposomes was analyzed by ABTS free radical cation decolorization and DPPH free radical scavenging assay. Data in Figure 4A showed that the ABTS radical scavenging rate of naked BLF was 40.46 ± 4.61%. BLF-Lip, CH-BLF-Lip and AL-CH-BLF-Lip showed higher antioxidant activity compared with naked BLF (60.30 ± 2.23%, 59.53 ± 2.6% and 54.65 ± 3.91%, respectively). It should be noted that the differences between the three BLF-loaded nanoliposomes were not significant. The DPPH free radical scavenging rate of BLF-Lip, CH-BLF-Lip and AL-CH-BLF-Lip on DPPH free radicals was 27.95 ± 2.1%, 33.59 ± 3.18%, and 36.77 ± 4.11%, respectively, which were significantly higher than naked BLF (18.32 ± 2.83%). Compared with BLF-Lip, the DPPH free radical scavenging ability was significantly improved after coating CH and AL (Figure 4B). The possible explanation is the formation or enhancement of hydrogen bonds during the encapsulation process and the synergistic effect of phospholipid, CH and AL [30]. In conclusion, BLF-loaded nanoliposomes can significantly improve the antioxidant capacity of BLF, especially after coating polysaccharides.

### 3.5. In Vitro Release Study

**Figure 5:**
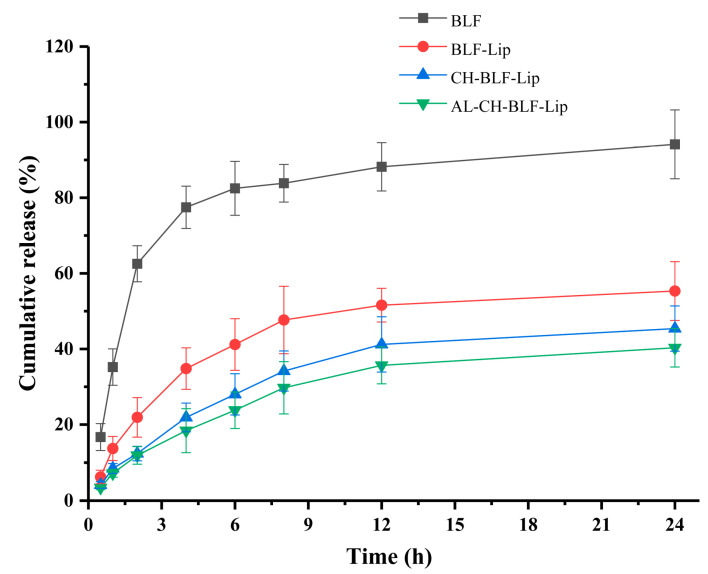
In vitro release behavior of naked BLF and BLF releasing from different BLF-loaded nanoliposomes. Data were presented as mean ± SD of three independent replications. BLF, bamboo leaf flavonoids; BLF-Lip, BLF-loaded nanoliposomes; CH-BLF-Lip, BLF-loaded chitosan coated nanoliposomes; AL-CH-BLF-Lip, BLF-loaded alginate-chitosan coated nanoliposomes.

Sustained release of nanoliposomes means that when nanoliposomes enter the body, the encapsulated compounds cannot be released quickly due to hydrogen bonding or the protective effect of liposome membrane, so they can last for a long time in the body, which is an important factor affecting their potential applications. In this study, an in vitro release model was used to partially reflect the prolonged residence time. The CR of naked BLF and BLF releasing from BLF-loaded nanoliposomes in dialysis bag was measured within 24 h (Figure 5). The CR ratio of naked BLF exceeded 60% after 2 h, while BLF-Lip, CH-BLF-Lip and AL-CH-BLF-Lip were only 21.92, 12.38 and 11.89%, respectively. After incubation for 24 h, naked BLF was almost completely released, and the CR ratio of BLF-Lip, CH-BLF-Lip and AL-CH-BLF-Lip were 55.33, 45.39 and 40.32%, respectively. Therefore, the polysaccharides coating can further prolong the release of BLF and achieve the effect of controlled release.

### 3.6. In Vitro Skin Permeation Study

**Figure 6:**
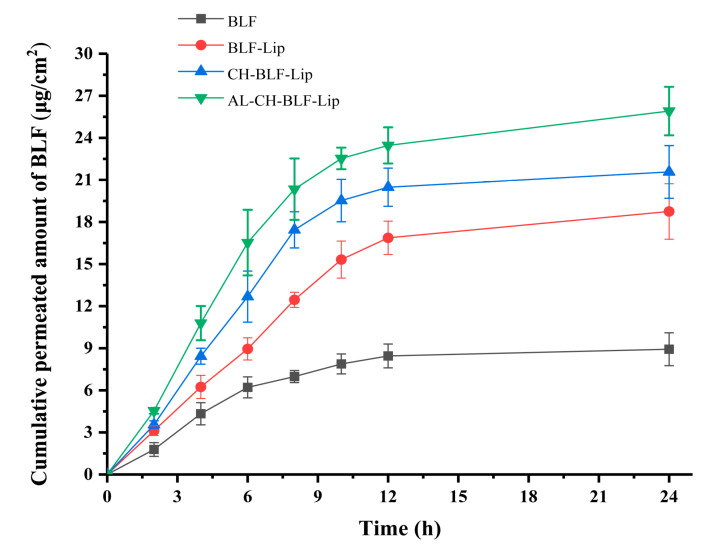
In vitro skin penetration of naked BLF and BLF releasing from different BLF-loaded nanoliposomes. Data were presented as mean ± SD of three independent replications. BLF, bamboo leaf flavonoids; BLF-Lip, BLF-loaded nanoliposomes; CH-BLF-Lip, BLF-loaded chitosan coated nanoliposomes; AL-CH-BLF-Lip, BLF-loaded alginate-chitosan coated nanoliposomes.

An in vitro skin penetration assay was performed to evaluate the transdermal efficiency of naked BLF and different BLF-loaded nanoliposomes. Mouse skin has similar barrier characteristics to human skin, and can be used as a substitute in in vitro transdermal studies [31]. The transdermal efficiency was calculated by the cumulative transdermal volume per cm^2^ (Figure 6). BLF and BLF-loaded nanoliposomes showed similar trends. After 6 h, the cumulative transdermal volume of naked BLF tended to be stable, while BLF-Lip, CH-BLF-Lip and AL-CH-BLF-Lip still increased significantly. The cumulative transdermal volume of BLF-Lip, CH-BLF-Lip and AL-CH-BLF-Lip after 24 h were 18.75 ± 1.98, 21.57 ± 1.88 and 25.91 ± 1.73 μg/cm^2^, respectively, which were significantly higher than naked BLF (8.93 ± 1.17 μg/cm^2^).

The stratum corneum is considered to be the main barrier to the transdermal delivery of compounds [32]. Liposomes can significantly improve the transdermal permeability of compounds, which may be due to the ability to mimic the cell membrane constituents. Generally, the smaller the particle size of the nanoparticles, the better the permeability [33,34]. However, in this study, CH-BLF-Lip and AL-CH-BLF-Lip with larger particle size showed better permeability, suggesting that there may be other factors affecting their transdermal absorption ability. One possible explanation is that CH and AL have good bioadhesivity, which can increase the contact time between the skin and nanoliposomes and promote the skin penetration ability of BLF [12]. The surface charge is also an important factor. Studies have shown that the greater the absolute value of zeta potential, the better the permeability [31]. In addition, negatively charged nanoliposomes promote skin penetration more effectively than positively charged nanoliposomes [12]. In summary, the result indicates that BLF-Lip and polysaccharide coated BLF-Lip have better skin permeability than naked BLF.

### 3.7. The Cytotoxicity of Naked BLF and Different BLF-Loaded Nanoliposomes

**Figure 7:**
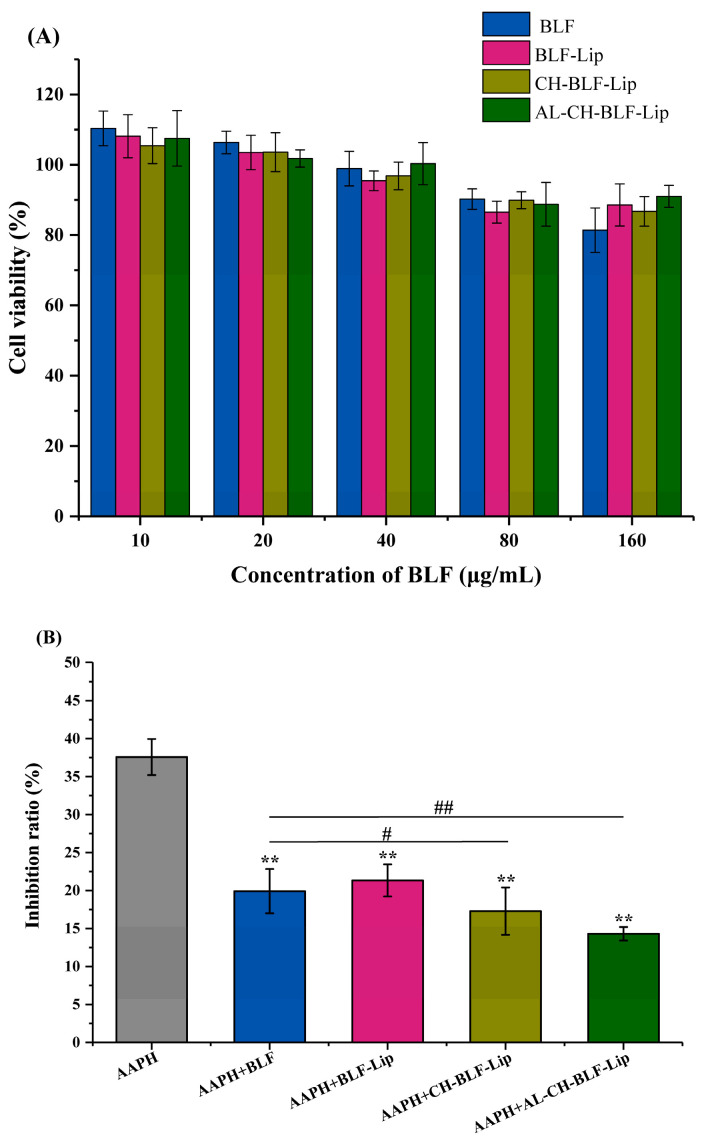
(**A**) The cytotoxicity at different concentrations (10–160 μg/mL BLF) of naked BLF and different BLF-loaded nanoliposomes in HaCaT cells. (**B**) The restoration effect of naked BLF and different BLF-loaded nanoliposomes (10 μg/mL BLF) on the inhibition of proliferation induced by AAPH in HaCaT cells. Data were presented as mean ± SD of three independent replications. ** *p* < 0.01 vs. AAPH group, # *p* < 0.05, ## *p* < 0.01 vs. AAPH + BLF group. BLF, bamboo leaf flavonoids; BLF-Lip, BLF-loaded nanoliposomes; CH-BLF-Lip, BLF-loaded chitosan coated nanoliposomes; AL-CH-BLF-Lip, BLF-loaded alginate-chitosan coated nanoliposomes; AAPH, 2,2′-Azobis (2-methylpropionamidine) dihydrochloride.

We further studied the cytotoxicity of naked BLF and BLF-loaded nanoliposomes in HaCaT cells at different BLF concentrations (10, 20, 40, 80, and 160 μg/mL). A cell viability above 90% indicates that the compound is non-toxic at the specified concentration [30]. The results showed that at the concentration of 10–80 μg/mL, naked BLF and BLF-loaded nanoliposomes had no cytotoxicity on HaCaT cells. When up to 160 μg/mL, the cell viability was reduced to 81.41%. However, encapsulation of BLF in nanoliposomes could reduce cytotoxicity (Figure 7A).

### 3.8. The Anti-Senescence Effect of BLF and BLF-Loaded Nanoliposomes

Next, we analyzed the inhibitory effect of naked BLF and BLF-loaded nanoliposomes on cellular senescence. Oxidative stress is a key factor in skin aging [35]. We used oxidant AAPH to treat HaCaT cells to establish a cellular senescence model [36].

#### 3.8.1. Assessment of Cell Proliferation

Impaired cell proliferation is an important characteristic of cellular senescence [37,38]. The results showed that 10 μg/mL BLF and BLF-loaded nanoliposomes significantly reduced the inhibitory effect of AAPH on the proliferation of HaCaT cells. The inhibition rate of BLF-Lip, CH-BLF-Lip and Al-CH-BLF-Lip on cell proliferation decreased from 37.58% to 21.33%, 17.29% and 14.31%, respectively. It is noteworthy that the effects of CH-BLF-Lip and AL-CH-BLF-Lip were significantly different from naked BLF, indicating that polysaccharides coating could better restore cell proliferation (Figure 7B).

#### 3.8.2. SAHF

**Figure 8:**
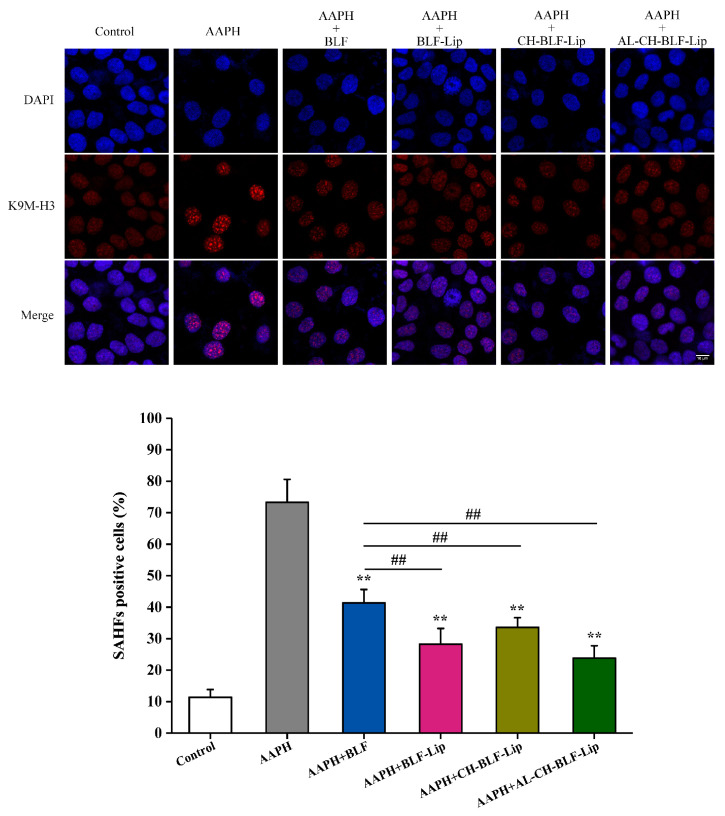
Representative images of SAHF formation. Scale bar = 10 μm. The percentage of SAHF positive cells was shown below. Data were presented as mean ± SD of three independent replications. ** *p* < 0.01 vs. AAPH group, ## *p* < 0.01 vs. AAPH + BLF group. BLF, bamboo leaf flavonoids; BLF-Lip, BLF-loaded nanoliposomes; CH-BLF-Lip, BLF-loaded chitosan coated nanoliposomes; AL-CH-BLF-Lip, BLF-loaded alginate-chitosan coated nanoliposomes; AAPH, 2,2′-Azobis (2-methylpropionamidine) dihydrochloride.

Cellular senescence is often accompanied by the appearance of punctate heterochromatin structure, known as SAHF, which can be visualized by staining with DAPI and antibody against K9M-H3 [38]. As shown in Figure 8, compared with the control group, 1 mM AAPH could significantly increase the proportion of K9M-H3 aggregated cells. After co-treatment with naked BLF and BLF-loaded nanoliposomes, the proportion of K9M-H3 aggregated cells decreased significantly (*p* < 0.01). Compared with naked BLF, the effects of BLF-Lip, CH-BLF-Lip and AL-CH-BLF-Lip were significantly different (*p* < 0.01).

#### 3.8.3. Expression of K9M-H3, p21 and p16 Proteins

**Figure 9:**
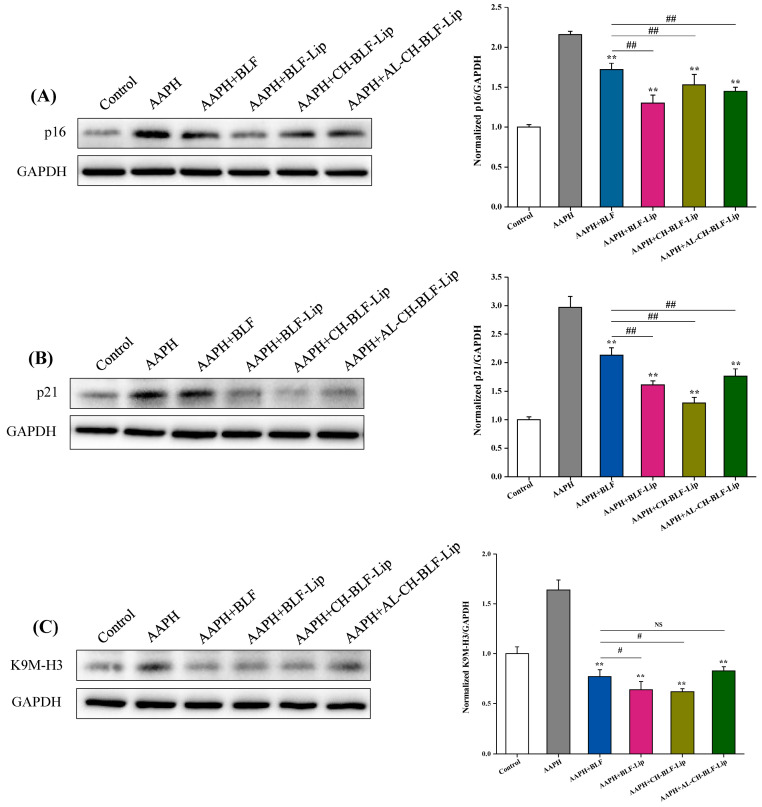
Protein expression of p16 (**A**), p21(**B**), and K9M-H3 (**C**) determined by western blotting. Quantitation was shown on the right. Data were presented as mean ± SD of three independent replications. ** *p* < 0.01 vs. AAPH treated group. # *p* < 0.05, ## *p* < 0.01 vs. AAPH + BLF treated group. NS, no significant difference.

Next, we detected the expression of K9M-H3 protein, and the results were shown in Figure 9. We found that after treating HaCaT cells with 1 mM AAPH, K9M-H3 protein not only aggregated, but also overexpressed. Naked BLF and BLF-loaded nanoliposomes could significantly reduce the expression level of K9M-H3 protein (*p* < 0.01). Compared with naked BLF, the effects of BLF-Lip and CH-BLF-Lip were significantly different (*p* < 0.05). Cell cycle regulators such as p16 and p21 are often used as biomarkers to detect senescent cells [38]. Naked BLF and BLF-loaded nanoliposomes could significantly reduce the overexpression of p16 and p21 proteins induced by AAPH in HaCaT cells. Compared with naked BLF, the effects of BLF-Lip, CH-BLF-Lip and AL-CH-BLF-Lip were significantly different (*p* < 0.01). In summary, BLF-loaded nanoliposomes have a stronger ability to inhibit cellular senescence than naked BLF.

## 4. Conclusions

In summary, based on positively charged CH and negatively charged AL deposition on the surface of BLF-Lip, this study has successfully prepared AL-CH-BLF-Lip. The increase of particle size, the positive and negative alternation of zeta potential, the increase of EE, the change of FTIR spectra and the increase of T_c_ and ΔH can prove this result. In addition, after encapsulating BLF in nanoliposomes, the antioxidant activity, in vitro release, skin permeation and anti-senescence ability have been significantly improved, especially CH-BLF-Lip and AL-CH-BLF-Lip. In short, alginate-chitosan coated nanoliposomes can be considered as effective delivery systems for improving the controlled release and skin permeability of BLF. In the future, BLF-loaded nanoliposomes can be used as potential therapeutics for skin aging, and the effectiveness needs to be verified by further clinical trials.

## Figures and Tables

**Table 1:** Characterization of BLF-loaded nanoliposomes. BLF, bamboo leaf flavonoids; BLF-Lip, BLF-loaded nanoliposomes; CH-BLF-Lip, BLF-loaded chitosan coated nanoliposomes; AL-CH-BLF-Lip, BLF-loaded alginate-chitosan coated nanoliposomes.

Liposomes	Particle Size (nm)	Polydispersity Index (PDI)	Zeta Potential (mV)	Encapsulation Efficiency (%)
BLF-Lip	152.13 ± 5.20	0.25 ± 0.06	−3.81 ± 0.79	71.31 ± 1.67
CH-BLF-Lip	194.63 ± 4.25	0.31 ± 0.04	8.43 ± 1.56	78.77 ± 1.59
AL-CH-BLF-Lip	228.90 ± 4.89	0.36 ± 0.03	−27.77 ± 0.45	82.74 ± 0.75

## Data Availability

Data is contained within the article and supplementary material.

## Supplementary Materials

The following supporting information can be downloaded at: https://www.mdpi.com/article/10.3390/antiox11051024/s1, Figure S1: The chemical structure of orientin, isoorientin, vitexin and isovitexin; Figure S2: Liquid chromatogram of bamboo leaf flavonoids prepared in our laboratory.
