# Supplementary material for: Alginate-Chitosan Coated Nanoliposomes as Effective Delivery Systems for Bamboo Leaf Flavonoids: Characterization, In Vitro Release, Skin Permeation and Anti-Senescence Activity
Source: Antioxidants (Basel). 2022 May 23;11(5):1024. doi: 10.3390/antiox11051024 (PMC9137723; doi:10.3390/antiox11051024)
Supplement: Supplementary file 1 [file antioxidants-11-01024-s001.zip › antioxidants-1712686-supplementary.pdf]

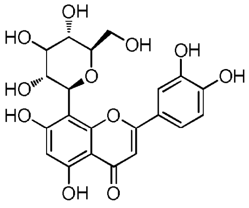

Orientin

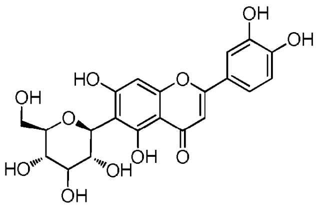

Isoorientin

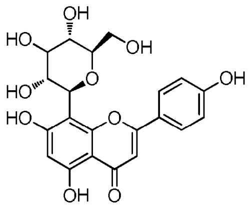

Vitexin

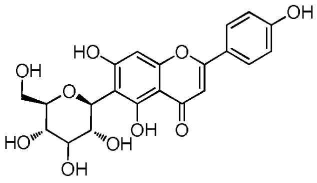

Isovitexin

Supplementary Figure S1 The chemical structure of orientin, isoorientin, vitexin and isovitexin.

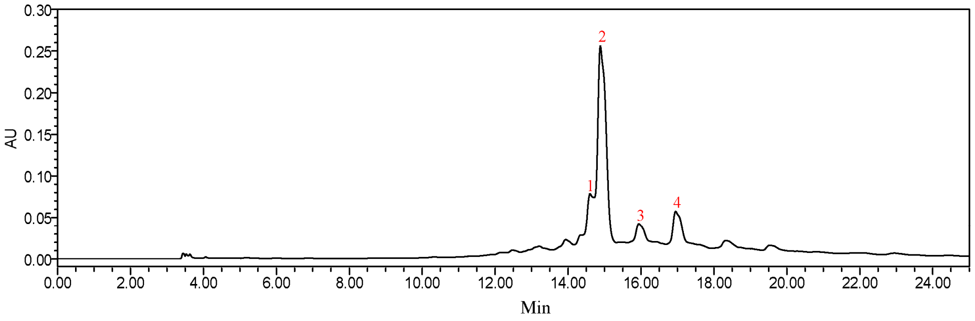

Peak	Flavonoid compound	Retention time	Area	Height	% Area
1	isoorientin	14.680	1028577	63027	10.64
2	orientin	14.965	3956320	214205	40.92
3	isovitexin	16.019	1511659	34918	15.63
4	vitexin	17.033	1302318	45940	13.47

Supplementary Figure S2 Liquid chromatogram of bamboo leaf flavonoids prepared in our laboratory.
